# ASO Author Reflections: Similar Short- and Long-Term Outcomes in Variable Use and Timing of Restaging MRI and Surgery After Preoperative Chemoradiotherapy for Rectal Cancer

**DOI:** 10.1245/s10434-019-07286-y

**Published:** 2019-03-07

**Authors:** Robin Detering, Pieter J. Tanis

**Affiliations:** Department of Surgery, Amsterdam UMC, University of Amsterdam, Amsterdam, The Netherlands

## Past

Preoperative chemoradiotherapy (CRT) has become standard of care in the treatment of patients with locally advanced rectal cancer. CRT results in downsizing facilitating complete resection and reduces the local recurrence rate, and 15–20% of patients achieve pathological complete response (pCR), which is associated with favorable oncological outcomes.^1^ However, one of the unresolved issues regarding preoperative CRT is the optimal time interval to total mesorectal excision (TME) surgery and the role of magnetic resonance imaging (MRI) restaging in clinical decision-making.

Studies have reported conflicting results regarding the association between the CRT–surgery interval and outcome parameters such as pathological response, specimen quality, margin status, and postoperative morbidity.^2^ Regarding MRI restaging, optimal methodology, timing, interpretation, and clinical relevance are still being discussed.

Therefore, the purpose of this cross-sectional study was to evaluate firstly variation in practice with respect to MRI restaging and its impact on time interval to surgery, and secondly the impact of timing of surgery on pathological, surgical, and long-term oncological outcomes.

## Present

At the population level, high use of restaging MRI after CRT with substantial variability in timing was observed in patients with locally advanced rectal cancer, which was not supported by guideline recommendations at that time.^3^ Also, substantial variability in the time interval between start of CRT and surgery was observed, partially related to differences in patient and tumor characteristics. The impact of MRI restaging on clinical decision-making was difficult to determine based on this retrospective study. Using the median CRT–surgery interval of 14 weeks as a cutoff, similar postoperative and long-term surgical and oncological outcomes were found for the two interval groups. This real-world evidence does not point towards an optimal CRT–surgery interval, but stresses the importance of a tailored approach within a multidisciplinary setting. The heterogeneity within locally advanced rectal cancer and the response to therapy questions the value of conducting randomized controlled trials (RCTs) like the GRECCAR-6 trial that aim to define just one optimal time interval.^2^

## Future

Nowadays, the impact of response evaluation by MRI restaging and timing of subsequent treatment after CRT would be different, aiming at identifying clinical complete responders who are candidates for a watch-and-wait policy.^4^ For the majority of patients still requiring (beyond) TME surgery after CRT, studies using standardized diffusion-weighted MRI restaging with structured MRI reporting at specified time intervals are needed to define the impact on clinical decision-making and subsequent outcome.^5^ One of the unresolved questions is whether the type of resection is influenced by MRI restaging. Is it safe to change from total exenteration to only “shaving” of the prostate, or from abdominoperineal resection to sphincter-saving surgery based on significant shrinkage of the tumor on MRI? Regarding CRT–surgery interval, patient randomization in future RCTs should be performed after MRI response assessment, in order to determine the impact of timing of surgery on margin status, morbidity, and oncological outcome within homogeneous subgroups of locally advanced rectal cancer.
